# U. S. V. M. A.

**Published:** 1896-05

**Authors:** 


### U. S. V. M. A.

THE officers of the Ohio State Veterinary Medical Association desire it to be known that it was the wish of the members that the question of meeting at Columbus, Ohio, or Buffalo, New York, be decided by vote, and that the change from Columbus to Buffalo could only be accomplished through a vote of the members filed with the Secretary. Of the nineteen members present at the last meeting of the State Association—all of whom were written to upon this point, and answers received by the Secretary from sixteen—fifteen voted to change from Columbus to Buffalo, therefore the semi-annual meeting will be held at Buffalo on September 1st, 2d, and 3d, in conjunction with the annual convention of the United States Association.

In the future all applicants for membership in the U. S. V. M. A. will be required to forward their initiation fee with the application. If the applicant is rejected the fee will be returned.

This year the reports of the State Secretaries will be of special interest, as instructions have been given to each one, on his acceptance of the office, directing the work of these officers in specific channels.

Already Detroit is in the field as a Western city for the meeting in 1897. As Kansas City launched its wish in this direction at Des Moines, there promises to be considerable rivalry for the place, particularly if California should enter the race again, and now that Treasurer Robertson has regained his wonted vigor, and no doubt will be with us at Buffalo in September, he may cling to his long-expressed wish that we shall cross the Rocky Mountains before the century ends, and thus have covered our whole country in reality as well as name.

There are rumors in the air that a closer allegiance to the code of ethics will be demanded of some of the members at the Buffalo meeting, and this should be so, or else the laws should be made elastic for all. Nothing breeds contempt for laws so much as ignoring their rigid enforcement with all who are amenable to them.

Dr. N. S. Mayo, of Kansas, will fill a place upon the programme for September next, the subject to be announced later.

The subject of tuberculosis will be allotted a place upon the programme and those aspects of the subject from the State's point of view, individually and collectively; the wisdom of compensation; interstate traffic; the influence of tuberculin; the adoption of compulsory police measures for its control; disinfection of premises, and those leading points only which are still unsettled and undetermined.

